# Co-overexpression of *AtSAT1* and *EcPAPR* improves seed nutritional value in maize

**DOI:** 10.3389/fpls.2022.969763

**Published:** 2022-09-15

**Authors:** Xiaoli Xiang, Binhua Hu, Zhigang Pu, Lanying Wang, Thomas Leustek, Changsheng Li

**Affiliations:** ^1^Institute of Biotechnology and Nuclear Technology, Sichuan Academy of Agricultural Sciences, Chengdu, China; ^2^The National Engineering Laboratory of Crop Stress Resistance Breeding, Anhui Agricultural University, Hefei, China; ^3^Department of Plant Biology, Rutgers University, New Brunswick, NJ, United States

**Keywords:** methionine, serine acetyltransferase1, 3′-phosphoadenosine-5′-phosphosulfate reductase, transcriptome profiling analysis, protein–protein interaction

## Abstract

Maize seeds synthesize insufficient levels of the essential amino acid methionine (Met) to support animal and livestock growth. *Serine acetyltransferase1* (*SAT1*) and *3′-phosphoadenosine-5′-phosphosulfate reductase* (*PAPR*) are key control points for sulfur assimilation into Cys and Met biosynthesis. Two high-MET maize lines *pRbcS:AtSAT1* and *pRbcS:EcPAPR* were obtained through metabolic engineering recently, and their total Met was increased by 1.4- and 1.57-fold, respectively, compared to the wild type. The highest Met maize line, *pRbcS:AtSAT1-pRbcS:EcPAPR*, was created by stacking the two transgenes, causing total Met to increase 2.24-fold. However, the *pRbcS:AtSAT1-pRbcS:EcPAPR* plants displayed progressively severe defects in plant growth, including early senescence, stunting, and dwarfing, indicating that excessive sulfur assimilation has an adverse effect on plant development. To explore the mechanism of correlation between Met biosynthesis in maize leaves and storage proteins in developing endosperm, the transcriptomes of the sixth leaf at stage V9 and 18 DAP endosperm of *pRbcS:AtSAT1*, *pRbcS:AtSAT1-pRbcS:EcPAPR*, and the null segregants were quantified and analyzed. In *pRbcS:AtSAT1-pRbcS:EcPAPR*, 3274 genes in leaves (1505 up- and 1769 downregulated) and 679 genes in the endosperm (327 up- and 352 downregulated) were differentially expressed. Gene ontology (GO) and KEGG (Kyoto encyclopedia of genes and genomes) analyses revealed that many genes were associated with Met homeostasis, including transcription factors and genes involved in cysteine and Met metabolism, glutathione metabolism, plant hormone signal transduction, and oxidation–reduction. The data from gene network analysis demonstrated that two genes, serine/threonine-protein kinase (CCR3) and heat shock 70 kDa protein (HSP), were localized in the core of the leaves and endosperm regulation networks, respectively. The results of this study provide insights into the diverse mechanisms that underlie the ideal establishment of enhanced Met levels in maize seeds.

## Introduction

Maize, like many other cereal crops, is one of the main nutritional resources for humans and livestock but lacks the essential amino acid methionine (Met). Met is a sulfur (S)-containing amino acid produced in three enzymatic steps from cysteine (Cys; Takahashi et al., 2011). Met is mainly accumulated in endosperm in Met-rich zein proteins, i.e., 18- and 10-kDa δ-zein and 15-kDa β-zein. The 18- and 10-kDa δ-zein make up less than 5% of the total zeins (Wu et al., 2009). The 15-kDa β-zein comprises 5–10% of total zeins (Thompson and Larkins, 1994). Met deficiency in the kernel is attributed to sulfur reduction in the leaves as a source and a low content of high-MET-containing endogenous proteins in seeds as a sink (Bagga et al., 2005; Wu et al., 2012).

Many approaches have been used to improve the nutritional quality of modern corn. Both the overexpression of 10-kDa δ-zein or knockdown of 22-kDaγ-zein significantly increase Met content but at the expense of other zein proteins and reduced Cys (Lai and Messing, 2002; Wu et al., 2012), indicating that the availability of S-amnio acids limits the total S-amino acids in zeins. Serine acetyltransferase (SAT) is an enzyme that catalyzes serine to form O-acetyl serine (OAS; Tabe et al., 2010). 3′-phosphoadenosine-5′-phosphosulfate reductase (PAPR) uses 3′-phosphoadenosine-5′-adenylylsulfate (PAPS) as the substrate and thioredoxins as the electron donor to form sulfite (Martin et al., 2005). 5′-adenylylsulfate (APS) reductase and PAPR constitute the overexpression line (Martin et al., 2005). Evidence from enzymological and physiological studies suggests that both reactions are key regulatory metabolic steps in the control of Cys synthesis. Assimilation into Cys occurs when sulfite reacts with OAS catalyzed by OAS thiolyase. In our previous study, a metabolic engineering approach leading to high MET with minimal or no perturbation to the plant phenotype was conducted by leaves specifically overexpressing these two key enzymes, SAT and PAPR. Met is increased 1.40- and 1.57-fold in maize seeds of *pRbcS:AtSAT1* (Xiang et al., 2017) and *pRbcS:EcPAPR* (Planta et al., 2017), respectively. In addition, 18- and 10-kDa δ-zein and 15-kDa β-zein protein in those lines also increased.

To further increase the Met level in maize kernels, the *pRbcS:AtSAT1* and *pRbcS:EcPAPR* lines were combined through crossbreeding to create a co-overexpression line. The 15-kDa β-zein and 18- and 10-kDa δ-zein content were about 1.5-fold higher than that of either overexpression line. The total Met levels were also much higher than in the single gene overexpression lines. However, the co-overexpression line displayed abnormal plant phenotypes and decreased yield.

Despite these advances in engineered maize with improved nutritional value, little is known concerning the underlying physiological and molecular mechanisms that cause sulfur overproduction in leaves and high-MET storage in the endosperm, which plays an important role in determining its economic and nutritional value.

RNA sequencing (RNA-Seq) in plants is an accurate, fast, and high-performance technology that is used to measure gene expression patterns, study gene function, and induce gene interactions. To explore the mechanism of Met production in leaf tissue, transport, and storage in maize, transcriptome profiling was performed using Null, *pRbcS:AtSAT1*, and *pRbcS:AtSAT1-pRbcS:EcPAPR* lines. Leaf samples were collected from the sixth leaf at stage V9, at which AtSAT1 and EcPAPR are highly expressed (Planta et al., 2017; Xiang et al., 2018). Endosperm tissues were collected 18 days after pollination (DAP) when protein bodies had a highly ordered architecture. The 18- and 10-kDa δ-zein are deposited in the center of the protein body. 15-kDa β-zein is located in the peripheral layer (Lending and Larkins, 1989). Here, the differentially expressed genes (DEGs) involved in MET synthesis, transport, and storage, nutrient reservoir, and plant abiotic stress response in the *pRbcS:AtSAT1* and *pRbcS:AtSAT1-pRbcS:EcPAPR* lines were summarized, which offers candidate genes for Met transport and storage in seeds for the following research. In addition, the contents of amino acids, zein and non-zein proteins were investigated in this study.

## Materials and methods

### Genetic materials

The *Arabidopsis thaliana serine acetyltransferase1* (*AtSAT1*, Gene Bank: BT008309.1) and *Escherichia coli 3′-phosphoadenosine-5′-phosphosulfate reductase* (*EcPAPR*, Gene Bank: NP_417242.1) were both driven by the maize Rubisco small subunit promoter (*pRbcS*, Gene Bank: AH005359.3). They were transferred to the maize B x A HiII hybrid separately and then crossed to “B73′’ for five generations to create B73 background OE lines. Both have been described separately elsewhere (Planta et al., 2017; Xiang et al., 2017). The obtained OE lines were referred to as *pRbcS:AtSAT1* and *pRbcS:EcPAPR*, respectively. *pRbcS:AtSAT1* and *pRbcS:EcPAPR* were then crossed to produce an *AtSAT1* and *EcPAPR* co-overexpression line, referred to as *pRbcS:AtSAT1-pRbcS:EcPAPR*. The analyzed lines (at least 15 individuals for each line), including *pRbcS:AtSAT1*, *pRbcS:EcPAPR*, *pRbcS:AtSAT1-pRbcS:EcPAPR*, Null segregant and high Met inbred line BSSS53 were grown in a field in Shanghai (N 31 11’, E 121 29’) in 2016 and 2017.

### Amino acid analysis

For leaf samples, the sixth leaf of V9 plants was ground to powder in liquid nitrogen; mature seeds with the embryo removed were ground into fine powder and passed through an 80-mesh sieve. The soluble and total amino acid content of the leaf and kernel samples were analyzed by Beijing Mass Spectrometry Medical Research Co. Ltd. (Beijing, China). The samples were pretreated with performic acid to yield acid-stable derivatives of Cys and Met, cystic acid, and methionine sulfoxide. The samples were acid hydrolyzed to yield the total amino acid content.

### Total zein and non-zein extraction

Total zein and non-zein proteins were extracted as previously described (Wu et al., 2012). For non-zein extraction, after removal of the supernatant, as described above, the solid remnant in the tube was washed with 1 mL zein extraction buffer three times to completely remove zein protein. The solid was evacuated for 30 min at 45°C (VAL model, Eppendorf) and then suspended in 1 mL non-zein extraction buffer [2% (vol/vol) 2-mercaptoethanol, 3.75 mM sodium borate (pH10), 0.3% SDS]. The mixture was maintained at 25°C for 2 h and then centrifuged at 15,700 × *g* (Eppendorf) for 10 min before 100 μl of the supernatant liquid was transferred into a new tube. Finally, 3 μl protein solution and 8 μl protein loading buffer were mixed and analyzed using SDS/PAGE [15% (wt/vol)] to determine the non-zein accumulation pattern. The gels were stained with Coomassie blue.

### Total protein measurement in mature dry seeds

The dry seeds were ground into a powder and passed through an 80-mesh sieve. Crude protein was measured using a modified Kjeldahl method (International, 1997) that determined the total nitrogen in nitrate-containing materials. The sample was digested in sulfuric acid; ammonia was then distilled, and excess acid was titrated. A conversion factor of 6.25 was used for feedstuffs. The total protein content in the dry seeds was calculated.

### Ribonucleic acid isolation and RNA-Seq analysis

Total RNA from the sixth leaves (V9 stage) of maize plants was extracted using TRIzol reagent (Invitrogen Inc., Waltham, MA) and purified with the RNeasy Mini Kit after DNase I digestion (Qiagen Inc., Germantown, MD, United States). Total RNA from 18 DAP endosperm was extracted using the same method as described for the leaves, except that endosperm RNA extract buffer was used before the TRIzol reagent. RNA-Seq experiments were conducted with RNA isolated from three biological replicates for the Null, *pRbcS:AtSAT1*, and *pRbcS:AtSAT1-pRbcS:EcPAPR* lines. Three plants from each genotype were mixed to comprise one biological replicate.

RNA integrity was evaluated using an Agilent 2100 Bioanalyzer (Agilent Technologies, Santa Clara, CA, United States). The samples with an RNA integrity number (RIN) ≥ 7 were subjected to subsequent analysis. The libraries were constructed using the TruSeq Stranded mRNA LTSample Prep Kit (Illumina, San Diego, CA, United States) according to the manufacturer’s instructions. The libraries were then sequenced on the Illumina sequencing platform (HiSeqTM 2500 or other platforms), and 150 bp/125bp paired-end reads were generated. Quality control was performed using FastQC (R). The paired-end reads were aligned to the B73 reference genome and the reference gene model dataset using TopHat/Bowtie2.^1^ The reference maize genome (RefGen_v4,^2^), transcript sequence,^3^ and gene model annotation files^4^ were downloaded from Ensemble Genomes.^5^ Gene expression levels in the Null, *pRbcS:AtSAT1*, and *pRbcS:AtSAT1-pRbcS:EcPAPR* lines were determined by normalized FPKM (fragment per kilobase of transcript (exon model) per million mapped reads) values (Mortazavi et al., 2008; Mizrachi et al., 2010). To determine the variation in expression between three replicates from the Null line and three replicates from *pRbcS:AtSAT1* and *pRbcS:AtSAT1-pRbcS:EcPAPR*, the absolute difference of the log_2_-fold change was calculated (*p*-value ≤ 0.05). Differentially expressed genes (DEGs) were identified using a false discovery rate (FDR) threshold.

### Gene-level quantification, analysis of differentially expressed genes, cluster analysis, gene ontology, and Kyoto encyclopedia of genes and genomes enrichment

The FPKM value of each gene was calculated using cufflinks (Trapnell et al., 2012), and the read counts of each gene were obtained by htseq-count (Anders et al., 2015). DEGs were identified using the DESeq functions estimateSizeFactors and nbinomTest (Anders, 2012). *p*-value < 0.05 and fold change > 2 or fold change < 0.5 were set as the thresholds for significant differential expression. Hierarchical cluster analysis of DEGs was performed to explore gene expression patterns. Gene ontology (GO) and Kyoto encyclopedia of genes and genomes (KEGG) pathway enrichment analyses of DEGs were performed using R based on the hypergeometric distribution (Team, 2014).

### Real-time quantitative reverse transcription-PCR

Total RNA extracted as described above was used for reverse transcription with the SuperScript III First Strand Kit (Invitrogen). quantitative reverse transcription-PCR (qRT-PCR) was performed as previously described. The following PCR program was used: 95°C for 120 s, followed by 40 cycles of 95°C for 5 s, 60°C for 30 s, and 95°C for 5 s, 65–95°C melt curve, increments of 0.5°C for 5 s. The gene expression levels relative to the maize *ZmActin* (Zm00001d012277) gene were analyzed using the 2^–ΔΔ^ ^Ct^ analysis method. The primers are listed in Supplementary Table 8.

### Weighted gene co-expression network analysis and module preservation analysis

Weighted gene co-expression network analysis (WGCNA; Langfelder and Horvath, 2008) based on the differentially expressed (DE) genes package in R was applied to construct a co-expression network using the expression values of 2425 DEGs from *pRbcS:AtSAT1-pRbcS:EcPAPR* leaves and endosperm upregulated DEGs. Clusters were obtained using the molecular complex detection (MCODE) algorithm. Degree centrality analysis of the co-expression network was performed to explore hub genes present in *pRbcS:AtSAT1-pRbcS:EcPAPR* leaves.

The protein interaction data were selected from the Search Tool for the Retrieval of Interacting Genes/Proteins (STRING) 9.1 database, and a network was constructed by linking sulfur reduction-related genes with the selected gene signatures using Cystoscope 3.1.0, which is a free software package for visualizing, modeling, and analyzing the integration of biomolecular interaction networks using high-throughput expression data and other molecular states (Shannon et al., 2003).

Subsequently, we investigated the substructure of the protein–protein interaction (PPI) network extracted from the above-constructed network and focused on highly connected nodes, known as clusters, using the MCODE (Bader and Hogue, 2003) clustering algorithm, which includes vertex weighting, complex prediction, and optional post-processing. The core-clustering coefficient was proposed as a metric for sorting the vertices in a graph with respect to their local neighborhood density. The software of the MCODE algorithm was obtained from http://baderlab.org/Software/MCODE. The highly interacting nodes in the clusters were identified by parameters keeping K-core = 4, node score cut-off = 0.3, and max depth up to 100.

## Results

### *pRbcS:AtSAT1-pRbcS:EcPAPR* co-overexpression line showed abnormal growth due to increasing sulfite assimilation

The *pRbcS:AtSAT1* maize transgenic line was created by overexpressing *AtSAT1* in maize leaves, driven by the leaf-specific bundle-sheath-specific promoter *RbcS1* (Xiang et al., 2017). The *pRbcS:EcPAPR* maize transgenic line was created using the same promoter to drive *EcPAPR* overexpression in maize, as described previously (Planta et al., 2017). The expression of high-MET protein 18- and 10-kDa δ-zein and 15-kDa β-zein was increased in the kernels of both transgenic lines described above. Therefore, the *pRbcS:AtSAT1* and *pRbcS:EcPAPR* maize transgenic lines were crossed to create the co-overexpression line (*pRbcS:AtSAT1-pRbcS:EcPAPR*).

**FIGURE 1:**
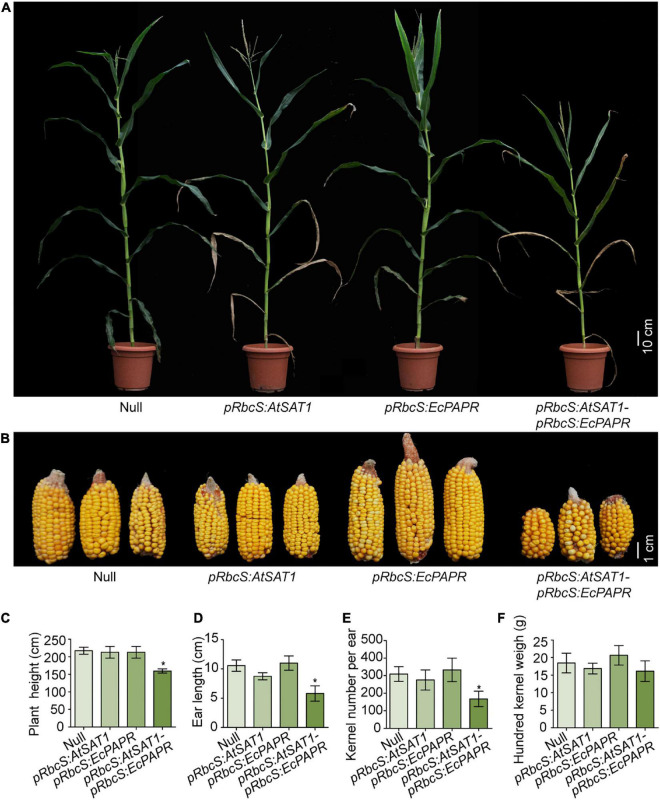
Perform‘ance of transgenic lines under field conditions. **(A)** Representative photo of flowering plants for each type as labeled in the photograph. **(B)** Representative photos of ears for each type as labeled in the photograph. **(C–F)** Comparison of plant height **(C)**, ear length **(D)**, kernel per ear **(E)**, hundred kernel weight **(F)** between Null, *pRbcS:AtSAT1*, *pRbcS:EcPAPR*, and *pRbcS:AtSAT1-pRbcS:EcPAPR*. The data shown are the means from 15 individual plants ± SD. Asterisks indicate significant differences from Null (Student’s *t*-test, *p-value < 0.05).

*pRbcS:AtSAT1* has dry leaf tips due to senescence (Xiang et al., 2017). *pRbcS:EcPAPR* shows no phenotypic abnormalities (Planta et al., 2017). We expected that the co-overexpression of *SAT* and *PAPR* would have little or no side effects on plant growth due to the increasing assimilation of sulfite. However, the *pRbcS:AtSAT1-pRbcS:EcPAPR* line showed severely stunted growth, chlorotic leaves, and early senescence at the V5 stage until harvest (Figure 1A). A similar phenotype was obtained by the co-overexpression of *PaAPR* and *EcPAPR* (Martin et al., 2005), but the phenotype described here was more severe than them. The size of the ears and the total kernel number decreased significantly (Figure 1B). In addition, the plant height (Figure 1C), ear length (Figure 1D), and kernel number per ear (Figure 1E) were also significantly decreased when compared with the Null, *pRbcS:AtSAT1*, and *pRbcS:EcPAPR* lines. However, the 100-kernel weight was not significantly different (Figure 1F).

### The *pRbcS:AtSAT1-pRbcS:EcPAPR* line had the highest free and protein-bound Met content in leaves and mature seeds

The *pRbcS:AtSAT1-pRbcS:EcPAPR* line inflated pools of free Cys and Met significantly more than *pRbcS:AtSAT1* and *pRbcS:EcPAPR* in the source leaves (Supplementary Figure 1A). Thus, *pRbcS:AtSAT1-pRbcS:EcPAPR* had a larger sulfur amino acid pool than the single gene overexpression lines *pRbcS:AtSAT1* and *pRbcS:EcPAPR*. The former line can supply more resources for seed sinks to synthesize sulfur-containing proteins. The contents of other free amino acids (AAs), such as Lys, Gly, Ile, Leu, Phe, and Trp, were increased in the *pRbcS:AtSAT1-pRbcS:EcPAPR* line. The contents of protein-bound Met and Cys were also significantly increased in *pRbcS:AtSAT1-pRbcS:EcPAPR* (Supplementary Figure 1B). In addition, the content of other AAs, including Lys, Gly, and Ile, showed similar increases among all high-MET lines.

**FIGURE 2:**
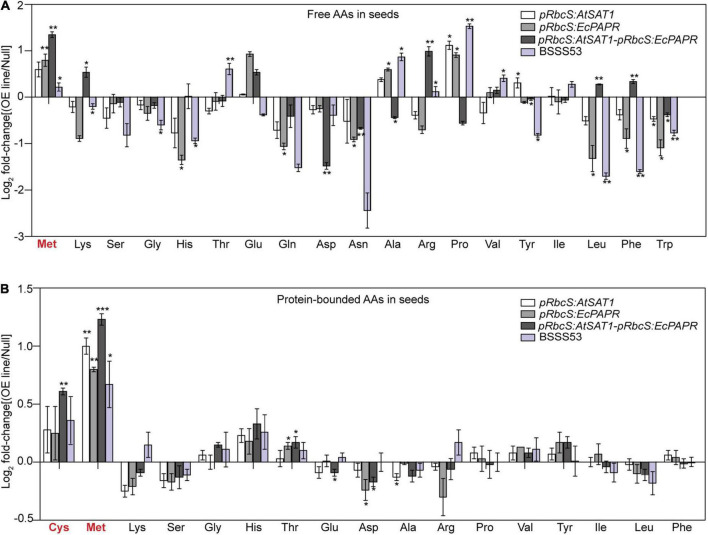
Foldchanges in free and protein-bound amino acid levels in transgenic seeds compared with Null. Data were log_2_-transformed and plotted in the bar graph. **(A)** Free amino acid levels. **(B)** Protein-bound amino acid levels. Bars to the left and right indicate a reduction and an increase, respectively, in the amino acid content of the *pRbcS:AtSAT1*, *pRbcS:EcPAPR*, *pRbcS:AtSAT1-pRbcS:EcPAPR*, and BSSS53 plants relative to Null. Student’s *t*-test at *p-value < 0.05 was used to determine the statistical significance of differences between the transgenic *pRbcS:AtSAT1*, *pRbcS:EcPAPR*, *pRbcS:AtSAT1-pRbcS:EcPAPR*, BSSS3, and non-transgenic Null kernels. Data shown are means ± SD of three replicates. **p-value ≤ 0.01 and ***p-value ≤ 0.001.

The composition of mature seeds represents the final result of gene expression throughout seed development (Tabe et al., 2010). To assess the effect of SAT1 and PAPR co-overexpression in the source leaves on maize seed composition at the end of maturation, free and protein-bound AAs were detected in hydrolyzed flour from pooled mature dry seed samples of Null, *pRbcS:AtSAT1*, *pRbcS:EcPAPR*, *pRbcS:AtSAT1-pRbcS:EcPAPR*, and BSSS53 lines, with three replicates. Maize inbred line BSSS53 is a high MET variant (Benner et al., 1989). Both free and protein-bound MET increased in the sink kernel, and the AA profile was changed to form a better nutrition pattern in the *pRbcS:AtSAT1-pRbcS:EcPAPR* line (Figures 2A,B). Free Lys, Arg, Leu, and Phe were increased in the *pRbcS:AtSAT1-pRbcS:EcPAPR* line compared with the Null and all other high-MET lines (Figure 2A). The major changes in the AA profile in the protein-bound AAs were increased Cys and Met, which were significantly higher in the *pRbcS:AtSAT1-pRbcS:EcPAPR* line (Figure 2B). The *pRbcS:AtSAT1-pRbcS:EcPAPR* line had the highest sulfur-AA content at 1.1% Cys and 4.46% Met.

### Kernel nutritional value improved in the *pRbcS:AtSAT1-pRbcS:EcPAPR* line without affecting the seeds’ total protein level

**FIGURE 3:**
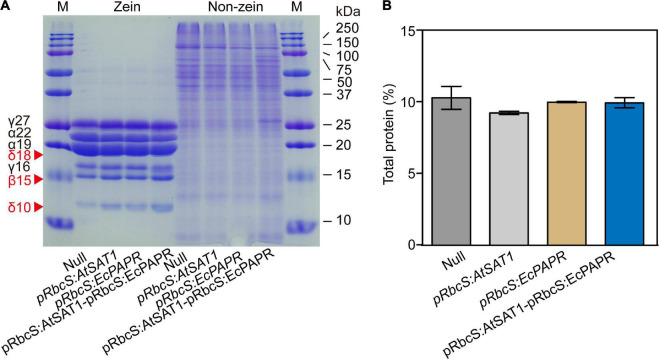
Zein and non-zein accumulation pattern in transgenic kernels. **(A)** Kernels from Null, *pRbcS:AtSAT1*, *pRbcS:EcPAPR*, and *pRbcS:AtSAT1-pRbcS:EcPAPR* were harvested from field plants. The kernels were fully mature, and protein profiles from three different kernels are shown. Protein from 300 μg dry weight of endosperm sample was loaded in each lane. The mass of each zein is indicated to the right of the figure. **(B)** Total protein of mature dry seeds.

In maize (*Zea mays*), the endosperm storage proteins zein and non-zein constitute a major protein component of the seed (Wu and Messing, 2010). Zein and non-zein of mature endosperm were analyzed by SDS-PAGE. Compared to the Null, all OE lines had higher expression of 18- and 10-kDa δ-zein and 15-kDa β-zein (Figure 3A). Moreover, the *pRbcS:AtSAT1-pRbcS:EcPAPR* line had the highest levels of 10-kDa δ-zein and 15-kDa β-zein and showed significantly higher expression levels than the single gene overexpression lines *pRbcS:AtSAT1* and *pRbcS:EcPAPR* (Figure 3A). The expression levels of 22- and 19-kDa γ-zein were slightly decreased in the *pRbcS:AtSAT1* and *pRbcS:AtSAT1-pRbcS:EcPAPR* lines. The 27- and 16-kDa γ-zein of the *pRbcS:AtSAT1-pRbcS:EcPAPR* line were not increased compared with either *pRbcS:AtSAT1* or *pRbcS:EcPAPR*. The non-zein portions of the Null, *pRbcS:AtSAT1*, and *pRbcS:EcPAPR* lines were not significantly different (Figure 3A). However, the non-zein content of *pRbcS:AtSAT1-pRbcS:EcPAPR* was slightly higher than that of the other three lines (Figure 3A), while the total protein in all lines was not significantly different (Figure 3B).

### Leaves of the high-MET line had 10.0-fold more differentially expressed genes than the endosperm

Transcriptomic analysis using the sixth leaves at the V9 stage and 18 DAP endosperm was carried out using Illumina RNA-Seq. About 80% of the raw reads from each sample were mapped to annotated gene-coding regions (Supplementary Table 1). Based on the global FPKM-expressing values (corrected *p*-value < 0.05), principal component analysis (PCA) showed that the biological replicates were clustered together, indicating experimental consistency. Hierarchical cluster analysis also showed a high correlation within sample replicates (Supplementary Figures 2A,B,E,F).

Compared to the leaf samples from the Null line, *pRbcS:AtSAT1* had 2158 DEGs (1327 up- and 831 downregulated genes; Supplementary Table 2). *pRbcS:AtSAT1-pRbcS:EcPAPR* had 3274 DEGs (1505 up- and 1769 downregulated genes; Supplementary Table 3). There were 1032 specific DEGs in *pRbcS:AtSAT1*, and 2148 DEGs in *pRbcS:AtSAT1-pRbcS:EcPAPR*; they shared 1126 common DEGs (Supplementary Figures 2C,D).

Compared to the endosperm samples from the Null line, *pRbcS:AtSAT1* had 307 DEGs (137 up- and 170 downregulated genes; Supplementary Table 4). *pRbcS:AtSAT1-pRbcS:EcPAPR* had 697 DEGs (327 up- and 352 downregulated genes; Supplementary Table 5). *pRbcS:AtSAT1* had 188 unique DEGs, and *pRbcS:AtSAT1-pRbcS:EcPAPR* had 460, while they shared 120 DEGs (Supplementary Figures 2C,D).

The *pRbcS:AtSAT1-pRbcS:EcPAPR* line contained more DEGs than the *pRbcS:AtSAT1* line, suggesting that the co-overexpression of *AtSAT1* and *EcPAPR* affects more genes than the *AtSAT1*-overexpression line. The number of DEGs in the leaf samples was 10-fold higher than in the endosperm, indicating that the leaf source could be the main limitation of the Met pool in the seed sink.

### Gene ontology and Kyoto encyclopedia of genes and genomes analysis revealed differentially expressed genes mainly enriched in the Met and glutathione pathway

**FIGURE 4:**
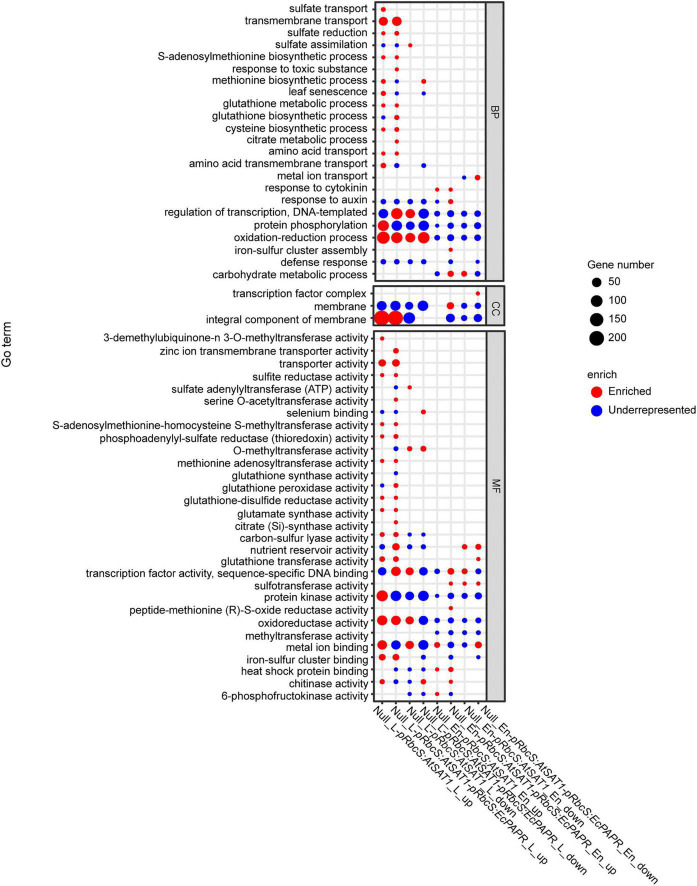
GO enrichment of DEGs across the comparisons. The Y-axis shows the GO term (correct *p*-value < 0.05). The *x*-axis indicates the enriched factor. The point sizes describe the enriched numbers in each term. The red color indicates enriched. The blue color indicates those that are underrepresented. (The figure was created by gg plot of the R language.)

To further reveal the functional roles of these DEGs involved in Met biosynthesis and storage. All up- and downregulated DEGs were used to investigate the GO annotations and in the KEGG pathway enrichment analysis (Supplementary Figures 3, 4, and 5). We further performed an enrichment analysis of sulfur-related GO terms (Figure 4). Upregulated genes enriched in both *pRbcS:AtSAT1* and *pRbcS:AtSAT1-pRbcS:EcPAPR* leaves included “sulfite reductase activity,” “methionine biosynthesis activity,” “glutamate synthase activity,” “cysteine biosynthetic process,” “amino acid transport,” “glutathione transferase activity,” and “sulfate reduction” (Figure 4). These indicated that both overexpression lines strengthened the expression pattern of sulfur-related pathway genes. For KEGG pathway enrichment of leaf tissue, many upregulated genes in both the *pRbcS:AtSAT1* and *pRbcS:AtSAT1-pRbcS:EcPAPR* lines were associated with “sulfur metabolism,” “cysteine and methionine metabolism,” and “glutathione metabolism” (Supplementary Figure 5). Up- and downregulated genes enriched in both *pRbcS:AtSAT1* and *pRbcS:AtSAT1-pRbcS:EcPAPR* leaves also included the “oxidation-reduction process,” indicating that the plant oxidation–reduction level changed in many aspects.

For KEGG pathway enrichment of endosperm tissue, upregulated genes in *pRbcS:AtSAT1* included “glutathione metabolism.” Upregulated genes in *pRbcS:AtSAT1-pRbcS:EcPAPR* included “biosynthesis of unsaturated fatty acid” and “fatty acid biosynthesis.” Downregulated genes in *pRbcS:AtSAT1-pRbcS:EcPAPR* included “peroxisome,” “nitrogen metabolism,” “cyanoamino acid metabolism,” “arginine and proline metabolism,” “carbon metabolism,” and “alanine, aspartate and glutamate metabolism” (Supplementary Figure 5).

### Met biosynthesis genes were upregulated in *pRbcS:AtSAT1-pRbcS:EcPAPR* leaves

The fold change of up- and downregulated DEGs in *pRbcS:AtSAT1-pRbcS:EcPAPR* was twice as high as the related DEGs in *pRbcS:AtSAT1* (Supplementary Tables 2–5). Upregulated genes related to sulfur metabolism, glutathione metabolism, amino acid transporters, and metal cation transporters are listed in Table 1. Several key regulatory genes involved in the sulfate reduction pathway were significantly upregulated in *pRbcS:AtSAT1-pRbcS:EcPAPR*, including *ATP sulfurylase1* (*APS1*), which adenylates sulfate (SO_4_^2–^) to form *5′-adenylylsulfate* (*APS*); *adenosine 5′-phosphosulfate reductase-like1* (*APRL1*) and *adenosine 5′-phosphosulfate reductase-like2* (*APRL2*), both of which catalyze sulfate to form sulfite (SO_3_^2–^); *sulfite reductase 1* (*SiR1*), which catalyzes sulfite to form sulfide (S^2–^); *serine acetyltransferase2* (*SAT2*), which catalyzes serine to form O-acetylserine (OAS); and S^2–^ reacts with OAS catalyzed by OAS thiol-lyase to form Cys (Table 1).

**TABLE 1:** Partial Up-regulated genes in *pRbcS:AtSAT1* and *pRbcS:AtSAT1-pRbcS:EcPAPR* leaves.

	Gene ID (v3)	*pRbcS:AtSAT1*		*pRbcS:AtSAT1-pRbcS:EcPAPR*		Description
			
		Fold change	*P*-value	Fold change	*P*-value	
Up-regulated S metabolism genes	Zm00001d021168	32.2	1.85E-18	79.6	1.46E-14	*UGT74F1 transfers UDP*
	Zm00001d020592	–	–	36.46	0.016549	*Glutelin-2*
	Zm00001d046226	7.4	3.03E-08	16.5	9.12E-11	*mrpa1 – multidrug resistance protein associated1*
	Zm00001d033981	–	–	2.3	0.0054	*aps1 – ATP sulfurylase1*
	Zm00001d048189	10.0	2.41E-07	–	–	*sulfate transporter 1;3 (SULTR1;3)*
	Zm00001d021596	–		4.4	2.26E-08	*aprl1 – adenosine 5′-phosphosulfate reductase-like1*
	Zm00001d006467	2.3	3.76E-03	3.5	7.43E-07	*aprl2 – adenosine 5′-phosphosulfate reductase-like2*
	Zm00001d038625	2.8	6.59E-12	5.4	7.61E-15	*sir1 – sulfite reductase1*
	Zm00001d028154	–	–	2.4	0.000149	*sat2- sat2 – serine acetyltransferase2*
Up regulated genes in GSH metabolism and metal transporters	Zm00001d035445	8.2	4.76E-09	11.1	3.20E-11	*gsh1 – gamma-glutamylcysteine synthetase1*
	Zm00001d010950	–	–	3.5	6.85E-07	*glutamate – cysteine ligase B, chloroplastic*
	Zm00001d043845	2.4	9.76E-09	2.3	1.24E-07	*glutamate synthase 1 [NADH], chloroplastic*
	Zm00001d024963	6.5	6.86E-06	11.6	4.60E-42	*gst22 – glutathione transferase22*
	Zm00001d042104	48.01	1.71E-22	102.2	3.97E-11	*gst7 – glutathione transferase7*
	Zm00001d018809	16.32	4.57E-07	37.4	1.36E-65	*gst6 – glutathione transferase6*
	Zm00001d024839	13.22	1.04E-16	34.3	6.55E-32	*gst2 – glutathione S-transferase2*
	Zm00001d042096	10.27	1.19E-14	25.7	3.92E-19	*gst21 – glutathione S-transferase21*
	Zm00001d029696	7.2	3.39E-05	19.0	5.35E-19	*gst34 – glutathione transferase34*
	Zm00001d034356	4.7	5.42E-17	11.7	6.48E-17	*gst5 – glutathione transferase5*
	Zm00001d029704	3.0	2.26E-09	5.5	2.28E-20	*gst37 – glutathione transferase37)*
	Zm00001d029708	7.1	8.90E-04	5.0	1.03E-10	*gst30 – glutathione transferase30*
	Zm00001d042225	–	–	4.4	9.79E-08	*heavy metal transport/detoxification protein*
	Zm00001d010410	–	–	2.4	8.93E-05	*metal cation transporter putative expressed*
	Zm00001d014569	–	–	2.6	6.03E-07	*ctap1-copper-transporting atpase p-type 1*
	Zm00001d015829	–	–	2.6	1.51E-06	*ctap2-copper-transporting atpase p-type 1*
	Zm00001d019228	4.2	6.84E-13	6.0	1.64E-13	*metal cation transporter*
	Zm00001d003616	–	–	2.41	3.20E-07	*glycolipid transporter activity*
	Zm00001d036965	10.84	2.76E-45	24.0	3.75E-33	*ZT4, Zinc transporter 4, metal cation transporter*
	Zm00001d034145	–	–	2.2	6.05E-05	*ZP1, Zinc-finger protein 1, salt tolerance protein*
	Zm00001d003195	–	–	3.1	0.058149	*STO, Salt tolerance protein (STO)*
	Zm00001d049954	5.1	3.82E-06	6.6	1.74E-10	*MDAR-Monodehydroascorbate reductase*
Up-regulated genes in MET metabolism and AAs transporters	Zm00001d049265			7.1	3.62E-09	*OMT-O-methyltransferase*
	Zm00001d040697	2.9	2.21E-09	3.4	0.000527	*SAMS3 S-adenosylmethionine synthetase*
	Zm00001d048060	2.1	5.12E-05	2.7	0.032008	*HMT-Homocysteine S-methyltransferase 1*
	Zm00001d042135	5.8	4.89E-08	11.8	3.48E-12	*AATT-amino acid transmembrane transporter*
	Zm00001d019225	3.9	4.67E-13	5.0	0.000173	*AAT-amino acid transporter*
	Zm00001d044533	–	–	2.5	6.29E-08	*transmembrane transport*
Up-regulated TFs	Zm00001d048681	–	–	836.7	3.69E-29	*gras84 – GRAS-transcription factor 84*
	Zm00001d048682	–	–	438.7	6.25E-27	*gras82 – GRAS-transcription factor 82*
	Zm00001d022442	–	–	6.0	0.104687	*bzip58 – bZIP-transcription factor 58*
	Zm00001d005208	–	–	5.6	8.79E-05	*nactf5 – NAC-transcription factor 5*
	Zm00001d034418	–	–	5.1	4.52E-06	*hagtf8 – GNAT-transcription factor 8*
	Zm00001d010399	–	–	4.3	1.68E-15	*wrky92 – WRKY-transcription factor 92*
	Zm00001d043491	12.31	5.86E-09	19.3	5.97E-22	*ereb134 – AP2-EREBP-transcription factor 134*
	Zm00001d039245	2.7	1.41E-07	5.0	7.09E-16	*wrky93 – WRKY-transcription factor 93*
	Zm00001d012527	2.9	1.16E-04	4.9	2.25E-06	*nactf23 – NAC-transcription factor 23*
	Zm00001d006001	2.3	6.18E-07	4.5	6.21E-13	*wrky71 – WRKY-transcription factor 71*
	Zm00001d034601	3.1	9.16E-09	4.3	4.81E-10	*nactf49 – NAC-transcription factor 49*

### Nutrient reservoir-related gene expression patterns were reformed in the *pRbcS:AtSAT1-pRbcS:EcPAPR* endosperm

*pRbcS:AtSAT1-pRbcS:EcPAPR* had more up- and downregulated DEGs than *pRbcS:AtSAT1* in 18 DAP endosperm (Supplementary Tables 4 and 5). The fold change in DEG expression in *pRbcS:AtSAT1-pRbcS:EcPAPR* leaves almost doubled compared to *pRbcS:AtSAT1*, while the endosperm values of these two high-MET lines were not significantly different. These results indicate that transgenic line *pRbcS:AtSAT1-pRbcS:EcPAPR* has a slightly greater effect on seed sink protein accumulation.

**FIGURE 5:**
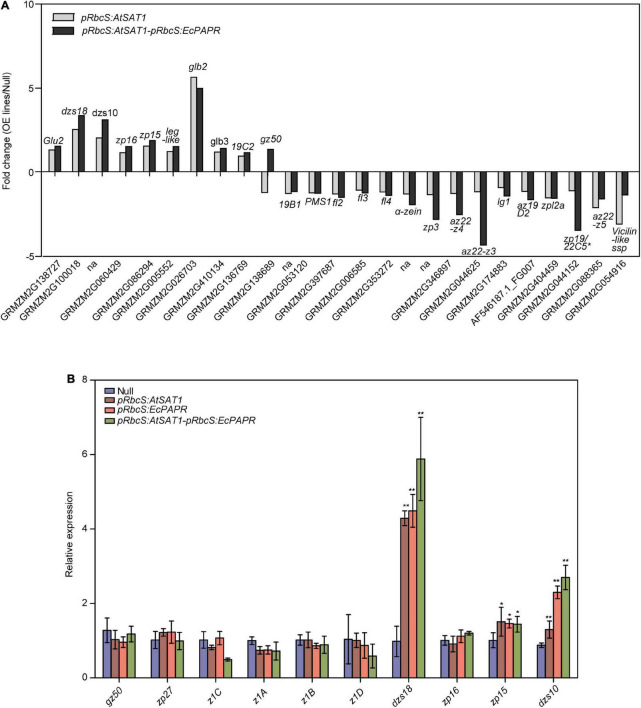
Comparison of gene expression patterns obtained using RNA-Seq and qRT-PCR. **(A)** Nutrient reservoir activity-related DEGs in *pRbcS:AtSAT1* and *pRbcS:AtSAT1-pRbcS:EcPAPR* compared with Null. Data were log_2_-transformed and plotted in the bar graph. **(B)** Expression of *gz50, zp27, z1C, z1A, z1B*,α*z1D, dzs18, zp16, zp15*, and *dzs10* in the OE lines relative to Null. The data are from three biological replicates per sample and are presented as mean ± SD. Asterisks indicate a significant difference from Null. (Student’s *t-*test, *p-value < 0.05 and ** *p*-value ≤ 0.001).

Previous results showed that *pRbcS:AtSAT1* increased MET-rich zein, 18- and 10-kDa δ-zein and 15-kDa β-zein, by increasing mRNA transcription levels (Xiang et al., 2017). The nutrient reservoir activity-related DEGs, including zein genes, were up- or downregulated in *pRbcS:AtSAT1* and *pRbcS:AtSAT1-pRbcS:EcPAPR* transcriptomic analysis (Figure 5A and Table 2). Most of the increased zeins were high-MET zeins, including *dzs18* (Zm00001d037436), which increased 2.51-fold in *pRbcS:AtSAT1* and 3.33-fold in *pRbcS:AtSAT1-pRbcS:EcPAPR*, and *dzs10* (Zm00001d045937), which was increased 2.00-fold in *pRbcS:AtSAT1* and 3.08-fold in *pRbcS:AtSAT1-pRbcS:EcPAPR*. Zein expression patterns showed similar results as those from the qRT-PCR of the Null, *pRbcS:AtSAT1*, *pRbcS:EcPAPR*, and *pRbcS:AtSAT1-pRbcS:EcPAPR* lines. The expression of *dzs18*, *dzs10*, and *zp15* in the *pRbcS:AtSAT1-pRbcS:EcPAPR* line was significantly improved (Figure 5B).

**TABLE 2:** List of nutrient reservoirs activity related DEGs.

id	Null vs *pRbcS:AtSAT1*		Null vs *pRbcS:AtSAT1-pRbcS:EcPAPR*		Description
			
	Fold change	*P*-value	Fold change	*P*-value	
Zm00001d020592	1.3	3.90E-01	1.5	6.84E-03	*Glutelin-2*
Zm00001d037436	2.5	2.42E-02	3.3	1.36E-06	*dzs18 – delta zein structural18*
Zm00001d045937	2.0	1.09E-01	3.1	1.87E-13	*dzs10*
Zm00001d005793	1.1	7.04E-01	1.5	8.53E-03	*zp16*
Zm00001d035760	1.5	2.49E-01	1.9	7.97E-05	*zp15 – zein protein, 15kDa*
Zm00001d011036	1.2	6.67E-01	1.5	3.67E-01	*legumin-like protein*
Zm00001d034413	5.6	3.23E-01	5.0	3.52E-01	*glb2 – globulin2*
Zm00001d038597	1.2	4.24E-01	1.4	2.35E-01	*glb3 – globulin3*
Zm00001d029782	–	–	1.1	4.61E-01	*19-kD*α*-zein (19C2)*
Zm00001d020591	–1.2	5.88E-01	1.3	2.31E-01	*gz50 – 50kD gamma zein*
Zm00001d048848	–1.3	1.34E-01	–1.1	3.45E-01	*19-kD*α*-zein (19B1)*
Zm00001d048850	–1.2	2.11E-01	–1.3	2.51E-01	α*-zein PMS1 precursor*
Zm00001d049243	–1.3	1.23E-01	–1.5	1.01E-01	*fl2 – floury2 (22kD*α*-zein 1*
Zm00001d009292	–1.1	7.92E-01	–1.2	5.66E-01	*fl3*
Zm00001d048851	–1.2	3.48E-01	–1.4	1.42E-01	*fl4 – floury4*
Zm00001d048816	–1.3	1.23E-01	–1.9	1.71E-05	α*-zein protein*
Zm00001d048817	–1.3	9.24E-02	–2.8	4.94E-08	*zp3 – zein protein3*
Zm00001d048812	–1.3	1.85E-01	–2.5	4.87E-04	*az22z4(az22z4 – 22kD alpha zein4)*
Zm00001d048809	–1.2	4.34E-01	–4.3	1.34E-06	*az22z3 – 22kD alpha zein3*
Zm00001d035700	–0.9	5.75E-01	–1.4	1.99E-02	*eg1 – legumin1*
Zm00001d030855	–1.1	7.28E-01	–1.6	1.65E-01	*az19D2 – alpha zein 19kDa D2*
Zm00001d048847	–1.5	1.13E-02	–1.6	2.82E-02	*zpl2a – zein polypeptidesL2a (zp12b)*
Zm00001d048813	–1.1	6.62E-01	–3.5	2.76E-05	zp19/22C5*(zein protein SF4C candidate 5)
Zm00001d048806	–2.1	3.22E-01	–1.9	4.79E-01	*az22z5 – 22kD alpha zein5*
Zm00001d029062	–3.1	1.14E-02	–1.3	5.40E-01	*Vicilin-like seed storage protein*
Zm00001d048818	–2.6	1.83E-02	–1.1	5.76E-01	unknown
Zm00001d049476	–1.5	4.64E-02	–1.7	3.98E-03	unknown
Zm00001d048176	–1.5	2.79E-01	–1.2	5.87E-01	unknown
Zm00001d048810	–1.6	1.51E-01	–3.3	9.86E-04	unknown
Zm00001d025059	–4.6	2.15E-03	–2.1	2.51E-01	unknown
Zm00001d048849	–1.3	2.10E-01	–1.1	4.63E-01	unknown
Zm00001d048852	–1.3	1.61E-01	–1.2	4.03E-01	unknown
Zm00001d019160	–1.7	3.17E-02	–1.5	5.67E-02	unknown
Zm00001d019162	–1.3	7.44E-01	–1.9	8.86E-02	unknown
Zm00001d048808	–3.5	4.19E-03	–1.4	6.44E-01	unknown
Zm00001d048219			–1.8	4.24E-01	*legumin-like protein*
Zm00001d004401			–4.6	1.04E-02	*germin-like protein subfamily 1 member 17*

* P≤0.05.

Upregulated transcription factors in endosperm mainly belong to EREB, bZIP, and HSFTF families (Table 3). Here, these TFs induced by increased MET flux into seeds might be involved in transcriptional or posttranscriptional regulation of *dzs18* and *dzs10* gene expression, thus affecting the 18- and 10-kDa δ-zein protein levels in the endosperm.

**TABLE 3:** TFs up regulated in 18 DAP endosperm of *pRbcS:AtSAT1* and *pRbcS:AtSAT1-pRbcS:EcPAPR.*

Up-down regulation	Gene ID (TF name or family)	
	
	*pRbcS:AtSAT1*	*pRbcS:AtSAT1-pRbcS:EcPAPR*
Up-regulated TFs in 18 DAP endosperm	Zm00001d019216 (*ereb64*)	Zm00001d019216 (*ereb64*)
	Zm00001d022461 (*ereb200*)	Zm00001d000179 (*ereb1*)
		Zm00001d024324 (*ereb54*)
		Zm00001d023332 (*wrky63*)
		Zm00001d043782 (*ereb126*)
		Zm00001d002762 (*ereb198*)
		Zm00001d026271 (*ereb205*)
	Zm00001d002143 (*bzip27*)	Zm00001d002143 (*bzip27*)
	Zm00001d009160 (*Trihelix*)	Zm00001d009160 (*Trihelix*)
	Zm00001d029506 (*lbd5*)	Zm00001d029506 (*lbd5*)
	Zm00001d038717 (*lbd33*)	
	Zm00001d010751 (*lbd38*)	
	Zm00001d052738 (*hsftf7*)	Zm00001d052738 (*hsftf7*)
	Zm00001d010812 (*hsftf16*)	Zm00001d026094 (*hstf20*)
	Zm00001d023669 (*naftf67*)	Zm00001d023332 (*heat shock*)
		Zm00001d043921 (*nactf82*)
		Zm00001d020492 (*wrky53*)
		Zm00001d022099 (*ca3p4*)
		Zm00001d028930 (*myb75*)
		Zm00001d017147 (*dbb6*)
		Zm00001d026536 (*C3H*)
		Zm00001d024200 (*CO-like*)
Down-regulated TFs in 18 DAP endosperm	Zm00001d041958 (*WRKY*)	Zm00001d041958 (*WRKY*)
	Zm00001d012757 (*GATA*)	Zm00001d012757 (*GATA*)
	Zm00001d026351 (*HD-ZIP*)	Zm00001d040651 (*ERF*)
	Zm00001d033215 (*NF-YA*)	Zm00001d039913 (*MADS54*)
	Zm00001d026447 (*ERF*)	Zm00001d031620 (*MIKC*)
	Zm00001d047967 (*bZip*)	Zm00001d040301 (*C3H*)
		Zm00001d021019 (*bHLH*)
		Zm00001d037098 (*bHLH*)
		Zm00001d017614 (*MIKC*)
		Zm00001d042560 (*LBD*)
		Zm00001d046755 (*ARR-B*)
		Zm00001d040362 (*Dof*)
		Zm00001d002718 (*GRAS*)
		Zm00001d015381 (*M-type*)
		Zm00001d041489 (*HD-ZIP*)
		Zm00001d030995 (*bZIP*)
		Zm00001d040536 (*bHLH*)

### PPI analysis of upregulated DEGs in *pRbcS:AtSAT1-pRbcS:EcPAPR* leaves and endosperm

**FIGURE 6:**
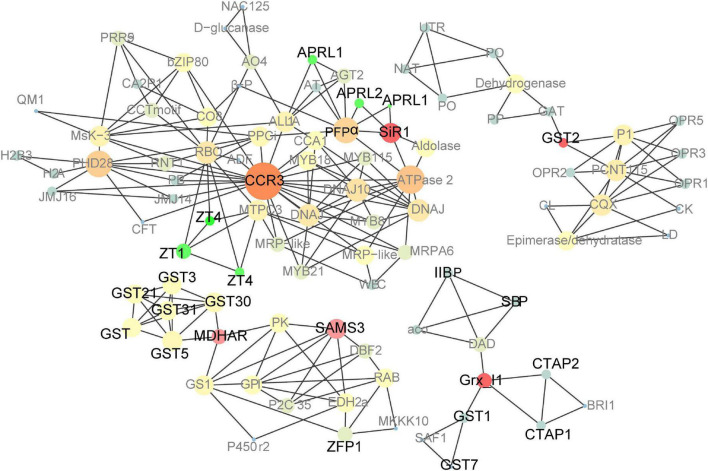
Co-expression network analysis of up-regulated genes in *pRbcS:AtSAT1-pRbcS:EcPAPR* mature leaves and 18 DAP endosperm. Cycle nodes represent genes, and the sizes of the nodes represent the power of the interrelation among the nodes. Edges between two nodes represent interactions between genes. The more edges of a gene, the more genes are connected to it, and the more central role it has within the network.

A large PPI network that included most of the genes was constructed, containing 601 nodes. The MCODE clustering algorithm was used to identify clusters in the PPI network. Using the MCODE plugin, four clusters (highly interconnected regions; Figure 6) in the networks were obtained with parameters set as follows: degree cut-off = 0.3, K-core = 4, and max depth = 100. A cluster is a complete n-node sub-graph, which means that within a sub-graph, each pair of nodes is connected by an edge (Zhuang et al., 2015). Detailed information for the gene symbols is listed in Supplementary Table 6. Most genes were upregulated in the leaves of these clusters. The sulfur reduction-related genes (*APRL1, APRL2*, and *SiR1*) and zinc transporters (*ZT1* and *ZTG4*) were clustered in one cluster; *CCR3* (*serine/threonine-protein kinase CCR3*) is the central hub gene, which has been identified as an enzyme that dependent on serine residues for its activity (Reddy and Rajasekharan, 2007). *MDHAR* (*monodehydroascorbate reductase*), *Grx_l1* (*glutaredoxin subgroup III*), and *GST2* (*glutathione transferase 2*) were the hub genes of the other three clusters.

### Identification of novel genes expressed in 18 DAP endosperm *via* PPI analysis of *pRbcS:AtSAT1-pRbcS:EcPAPR* up- and downregulated differentially expressed genes

**FIGURE 7:**
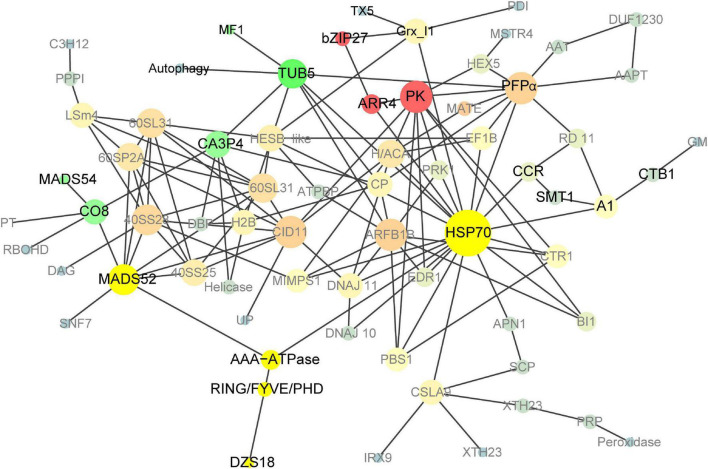
Co-expression network analyses of DEGs in *pRbcS:AtSAT1-pRbcS:EcPAPR* 18 DAP endosperm. Cycle nodes represent genes; the size of nodes represents the power of the interrelation among the nodes, and edges between two nodes represent interactions between genes. The more edges of a gene, the more genes are connected to it, and the more central role it has within the network.

To further explore the protein interaction in the 18 DAP endosperm of *pRbcS:AtSAT1-pRbcS:EcPAPR*, we applied a protein network-based approach to identify subnetworks that may provide new insights into the functions of pathways involved in high-MET storage protein rather than single genes. This reflects the aggregating behavior of genes connected in a PPI network (Goel et al., 2018). The network was binary, and all interactions were unweighted and undirected. A graph that included the majority of the genes containing 69 nodes was constructed (Figure 7) based on our analysis. The details of the gene symbols in the network are listed in Supplementary Table 8. The size of each node represents the degree index. The degree of its nodes indicates the number of interactions of a single node with all the other nodes. The top 10 hub genes included *HSP70* (*70-kDa heat shock proteins*), *PK* (*Pyruvate kinase*), *ARFB1B* (*ADP-ribosylation factor B1B*), *PFP*α (*Pyrophosphate – fructose 6-phosphate 1-phosphotransferase alpha subunit*), *CID11* (*CTC-interacting domain 11, RNA-binding protein*), *MADS52* (*MADS transcription factor 52*), *40SS28* (*40S ribosomal protein S28*), *TUB5* (*beta tubulin5*), *60SL31* (*60S ribosomal protein L31*), and *60SP2A* (*60S acidic ribosomal protein P2A*). The results revealed that *HSP70* was the centrality (hub) gene of the network, which functions actively in many aspects, such as protein folding, unfolding, regulation, targeting, aggregation and disaggregation, homeostasis, and degradation (Fernandez-Fernandez et al., 2017; Fernandez-Fernandez and Valpuesta, 2018; Rosenzweig et al., 2019).

## Discussion

### Overproduced sulfur-related compounds in *pRbcS:AtSAT1-pRbcS:EcPAPR* leaves had negative effects on plant growth

Increased Met and Cys in *pRbcS:AtSAT1-pRbcS:EcPAPR* indicated a higher sulfur content in the plants than wild type. Increased S reduction has profound consequences for the synthesis of all S-metabolites, including sulfur amino acids Met, Cys, and especially GSH and changed cell processes, such as oxidation–reduction, amino acid transport, and TF expression levels. The *pRbcS:AtSAT1-pRbcS:EcPAPR* plants grew slowly, were weaker, and had less biomass, smaller ears, and fewer kernels per ear, which is similar to *PaAPR* and *EcPAPR* co-overexpression lines (Martin et al., 2005).

From our transcriptome analysis, this might occur because S overproduction causes leaf cells to overaccumulate reaction sulfur species (RSS) in plants. RSS in the form of persulfidated cysteines (Cys-S-S) is produced endogenously and co-translationally introduced into proteins (Olson, 2020). *pRbcS:AtSAT1-pRbcS:EcPAPR* had plant cellular damage and cell component changes due to high levels of S-compounds, such as RSS. Oxidative stress induced by RSS can lead to cell death and tissue injury (Foyer and Noctor, 2011; Olson, 2021). Plants have evolved antioxidant mechanisms to deal with RSS, in which many genes, such as *S-transferase genes* (*GSTs*), ascorbate peroxidase genes, and glutaredoxins (GRXs), are involved (Xie et al., 2018). In this study, several *GST* genes were upregulated in *pRbcS:AtSAT1-pRbcS:EcPAPR*, such as *GST2*, *GST5*, *GST6*, *GST7*, *GST21*, *GST22*, *GST30*, *GST34*, and *GST37*, which mainly catalyze the conjugation of GSH onto xenobiotics, and some have GSH-dependent peroxidase activity against H_2_O_2_, H_2_S, and organic peroxides (Dixon et al., 2009; Tossounian et al., 2018; Olson, 2020). Several GST-encoding genes are strongly induced by oxidative stress (Wachter et al., 2005), thus helping plants cope with biotic and abiotic stress. This indicates that RSS induced by sulfur overproduction may be the main reason for stunting and dwarf phenotypes in *pRbcS:AtSAT1-pRbcS:EcPAPR* plants.

RSS signals *via* oxidation reactions with protein cysteine sulfur, and they produce identical effector responses, such as GSH. GSH can modulate reactive oxygen species by oxidizing the cysteine residues of transcription factors and signaling molecules (Lu, 2009). Many genes related to GSH synthase were upregulated in *pRbcS:AtSAT1-pRbcS:EcPAPR*, including *Glutamate synthase 1* (*GS1*), which is the key enzyme in N assimilation involved in glutamine synthesis (Seger et al., 2015); *glutamate-cysteine ligase B* (*GCL*), which catalyzes GSH biosynthesis (Musgrave et al., 2013); and *gamma-glutamylcysteine synthetase1* (*GSH1*), which is conjugated with CYS to form γ-glutamycsteine (Lu, 2013). GSH1 overexpression has a negative effect on tobacco growth (Creissen et al., 1999). The negative effects of GSH accumulation also explain this phenotype (Martin et al., 2005).

### Met production in maize leaves and transport to seeds

Several genes related to Met metabolism were upregulated in *pRbcS:AtSAT1-pRbcS:EcPAPR*, including *Homocysteine S-methyltransferase 1* (*HMT1*), which catalyzes SMM to be reconverted to Met (Mudd and Datko, 1990); *S-adenosylmethionine synthetase* (*SAMS3*), which is formed by MET adenosylation and is the precursor plant of certain polyamines and the plant hormone ethylene (Roje, 2006); and *O-methyltransferase* (*OMT*), which is dependent on *S*-adenosyl-l-methionine and can catalyze a variety of secondary metabolites (Liu et al., 2018). Some amino acid transporter genes were upregulated in both the *pRbcS:AtSAT1* and *pRbcS:AtSAT1-pRbcS:EcPAPR* leaves (Table 1).

**FIGURE 8:**
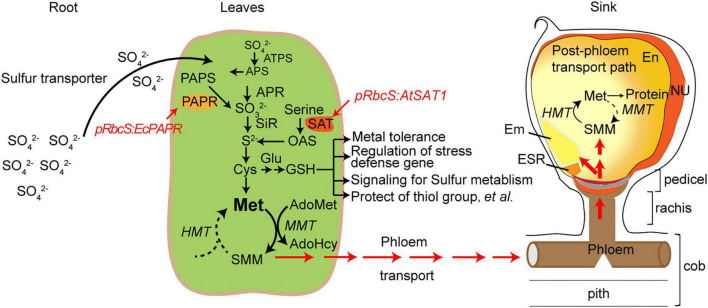
Schematic diagram of sulfur pools in developing maize seeds. SO_4_^2–^, sulphate; SO_3_^2–^, sulfite; S^2–^, sulphide; ATP-S, ATP-sulfurylase; APS, adenosine 5′- phosphosulfate; APR, APS reductase; SiR, sulfite reductase; SAT, serine acetyltransferase; OAS-TL, O-acetyserine(thiol)lyase; OAS, O-acetylserine; PAPR, 3′-phosphoadenosine-5′-phosphosulfate reductase; PAPS, 3’-phosphoadenosine-5’-phosphosulfate; Cys, cystenine; Glu, glutamate; GSH, reduced glutathione; Cys, cysteine; Met, methionine; HMT: Homocysteine S-methyltransferase, SMM, S-methylmethionine; MMT, methionine S-methyltransferase; AdoMet, S-Adenosyl-l-methionine; AdoHcy, S-adenosylhomocysteine; En, endosperm; Nu, nucellus; ESR, embryo-surrounding region; and BETL, basal endosperm transfer cell layer.

S reduction can be engineered by overexpressing the key enzymes in the pathway and increasing Met in the seeds (Martin et al., 2005; Wu et al., 2012; Planta et al., 2017; Xiang et al., 2017). However, Met has a long journey to arrive at the seeds. Met is converted into S-methylmethionine (SMM) and arrives in the seeds in SMM form *via* the SMM cycle in the leaves and seeds (Figure 8; Mudd and Datko, 1990). Sulfur arrives in the seeds as GSH and SMM. SMM is recycled to Met for use in protein synthesis (Brunold and Rennenberg, 1997; Bourgis et al., 1999). Therefore, in addition to S reduction, there are other limitations causing MET deficiency in seeds; one factor is amino acid transporters that transport AAs from leaf tissue through vacuoles to the seed sink.

The transport of amino acids into plant cells is known to involve a range of proton-coupled symporters with various substrate specificities, some of which overlap (Rentsch et al., 1998). To date, SMM appears not to have been tested as a substrate. *A priori*, it could be a substrate for the known general amino acid or basic amino acid transporter (Chen and Bush, 1997; Rentsch et al., 1998); alternatively, there could be an SMM-specific transporter similar to the SAM permease of yeast (Rouillon et al., 1999). Several AA transporters were increased in the *pRbcS:AtSAT1* and *pRbcS:AtSAT1-pRbcS:EcPAPR* lines, including *AAT* (amino acid transporter, Zm00001d019225) and *AATT* (amino acid transmembrane transporter, Zm00001d042135; Table 1). Identifying the transporter(s) responsible for phloem loading of SMM is of particular interest, given that phloem loading could play an important role in determining the composition of phloem sap and hence the amino acid content and nutritional value of seed storage proteins (Chen and Bush, 1997).

Therefore, engineering the SMM-related transporter would also increase the MET flux into the seeds. The combination of S reduction key enzymes and SMM transporters is another approach for super-high MET maize breeding.

### Transcription factors involved in stress response and maize seeds’ nutritional value

GSH, together with phytochelatins, is known to contribute to heavy metal detoxification and a range of stress responses (Cobbett, 2000; Lu, 2013). Many heavy metal-related genes were upregulated in *pRbcS:AtSAT1-pRbcS:EcPAPR*, including *Zinc transporter 4* (*ZT4*), which may play a crucial role in avoiding Zn stress in the root meristematic tissue before activation of Zn export systems and syntheses of phytochelatins (Kawachi et al., 2009); and *copper-transporting ATPase p-type 1* (*CTAP1*), which has a homolog heavy metal *ATPase 1* (*HMA1*) in Arabidopsis that is a Cu-ATPase functioning to import Cu into chloroplasts (Williams and Mills, 2005; Seigneurin-Berny et al., 2006). The enzyme functions as a pump for heavy metals, including Zn, Co, Cd, Pb, and Cu (Moreno et al., 2008) and is also involved as a metal sequestration transporter that contributes to Zn detoxification by reducing Zn content in plastids (Kim et al., 2009). Copper-transporting *ATPase p-type 2* (*CTAP2*), which has a homolog heavy metal *ATPase 5* (*HMA5*) in Arabidopsis, is also involved in Cu detoxification. Many stress response genes were upregulated in *pRbcS:AtSAT1-pRbcS:EcPAPR*, including *Zinc-finger protein 1* (*ZF1*), which is a salt-tolerant zinc finger protein. Transcription factors in the gibberellin (GA) signaling pathway were upregulated at a very high level; these included *GRAS84* and *GRAS82* transcription regulators, which increased 836.0- and 438.0-fold, respectively (Table 1). These regulators are known as *SCL14* (*scarecrow-like 14*), *DELLAs* (*a family of nuclear growth-restraining proteins that mediate the effect of the phytohormone gibberellin on growth*), and *SHR/SCR* (*SHORT-ROOT/SCARECROW*)in *Arabidopsis*, and they interact with the *TGA2* (AHBP-1b), an Arabidopsis bZIP transcription factor and thus affect the transcription of stress-responsive genes (Ralf Weigel et al., 2002; Heo et al., 2011).

Transcription factors play vital roles in maize kernel development. Here, we identified 20 up- and 17 downregulated TFs in 18 DAP endosperm (Table 3), including NAC, bZIP, GRAS, WRKY, AP2/EREB, MADS, and HSF. Among these TF families, *ZmbZIP22* regulates 27-kDa γ-zein (Li et al., 2018), and total Met in *Zmbzip22* mutants increases by 3.4% (Mach, 2018). *ZmMADS47* regulates 50-kD γ-zein (Xie et al., 2018). Little is known about 15-kDa β-zein and 18- or 10-kDa δ-zein regulation. The only information for *dzs10* regulation is a *Zpr10/(22)* factor on chromosome 4, which is a post-transcriptional regulator of zein accumulation in maize inbred line BSSS53 (Fisher, 1991; Chaudhuri and Messing, 1994). *Dzs18* shares a high sequence similarity with *dzs10*, indicating that they may have similar regulation patterns. High-MET zein dzs18 is directly connected with ING/FYVE/PHD (ING/FYVE/PHD zinc finger superfamily protein) and indirectly connected with AAA-ATPase, MADS52, and HSP70 (Figure 7 and Table 2), indicating that these related genes are candidates for uncovering 18-kDa δ-zein synthesis and storage patterns. Up- or downregulated TFs induced by increased MET content in endosperm in Table 3 also provide references for uncovering the transcriptional regulation mechanism of high-MET zein genes (15-kDa β-zein and 18- or 10-kDa δ-zein).

## Data availability statement

The datasets generated for this study can be found in Genome Sequence Archive (GSA) in National Genomics Data Center (NGDC) database with the accession number: PRJCA010079, available at https://ngdc.cncb.ac.cn/search/?dbId=gsa&q=PRJCA010079.

## Author contributions

CL and XX was conceived and designed by the experiment. BH, ZP, and LW collected the phenotypes and performed the data analysis. XX and CL was written by the manuscript. LT was edited the manuscript. All authors have read and approved the manuscript.

## Footnotes

1
http://ccb.jhu.edu/software/tophat/

2
http://ftp.ensemblgenomes.org/pub/plants/release-34/fasta/zea_mays/dna/Zea_mays.AGPv4.dna.toplevel.fa.gz

3
http://ftp.ensemblgenomes.org/pub/plants/release-34/fasta/zea_mays/cdna/Zea_mays.AGPv4.cdna.all.fa.gz

4
http://ftp.ensemblgenomes.org/pub/plants/release-34/gff3/zea_mays/Zea_mays.AGPv4.34.gff3.gz

5
http://ensemblgenomes.org/
