# Supplementary material for: Co-overexpression of AtSAT1 and EcPAPR improves seed nutritional value in maize
Source: Front Plant Sci. 2022 Sep 15;13:969763. doi: 10.3389/fpls.2022.969763 (PMC9520583; doi:10.3389/fpls.2022.969763)
Supplement: Supplementary file 1 [file Data_Sheet_1.PDF]

Supplementary Material

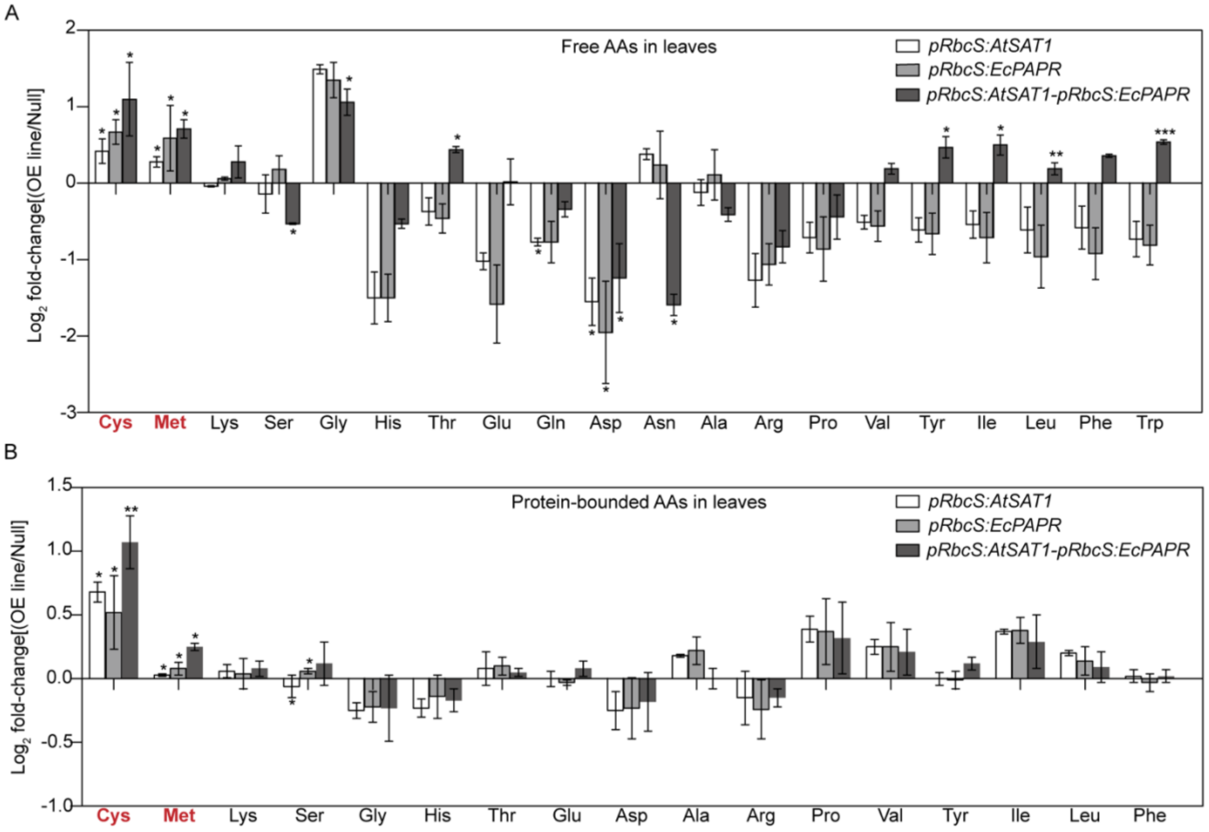

Figure S1 Foldchanges in free and protein-bounded amino acid levels in transgenic leaves compared with Null.

(A) Free amino acid levels. (B) Protein-bound amino acid levels. Data were $\log_2$-transformed and plotted in the bar graph. Bars to the left and right indicate a reduction and an increase, respectively, in the amino acid content of the *pRbcS:AtSAT1*, *pRbcS:EcPAPR*, and *pRbcS:AtSAT1-pRbcS:EcPAPR* plants relative to Null. Student's t test at **p*-value < 0.05 was used to determine the statistical significance of differences between the transgenic *pRbcS:AtSAT1*, *pRbcS:EcPAPR*, *pRbcS:AtSAT1-pRbcS:EcPAPR*, and non-transgenic Null kernels. Data shown are means ± SD of three replicates.

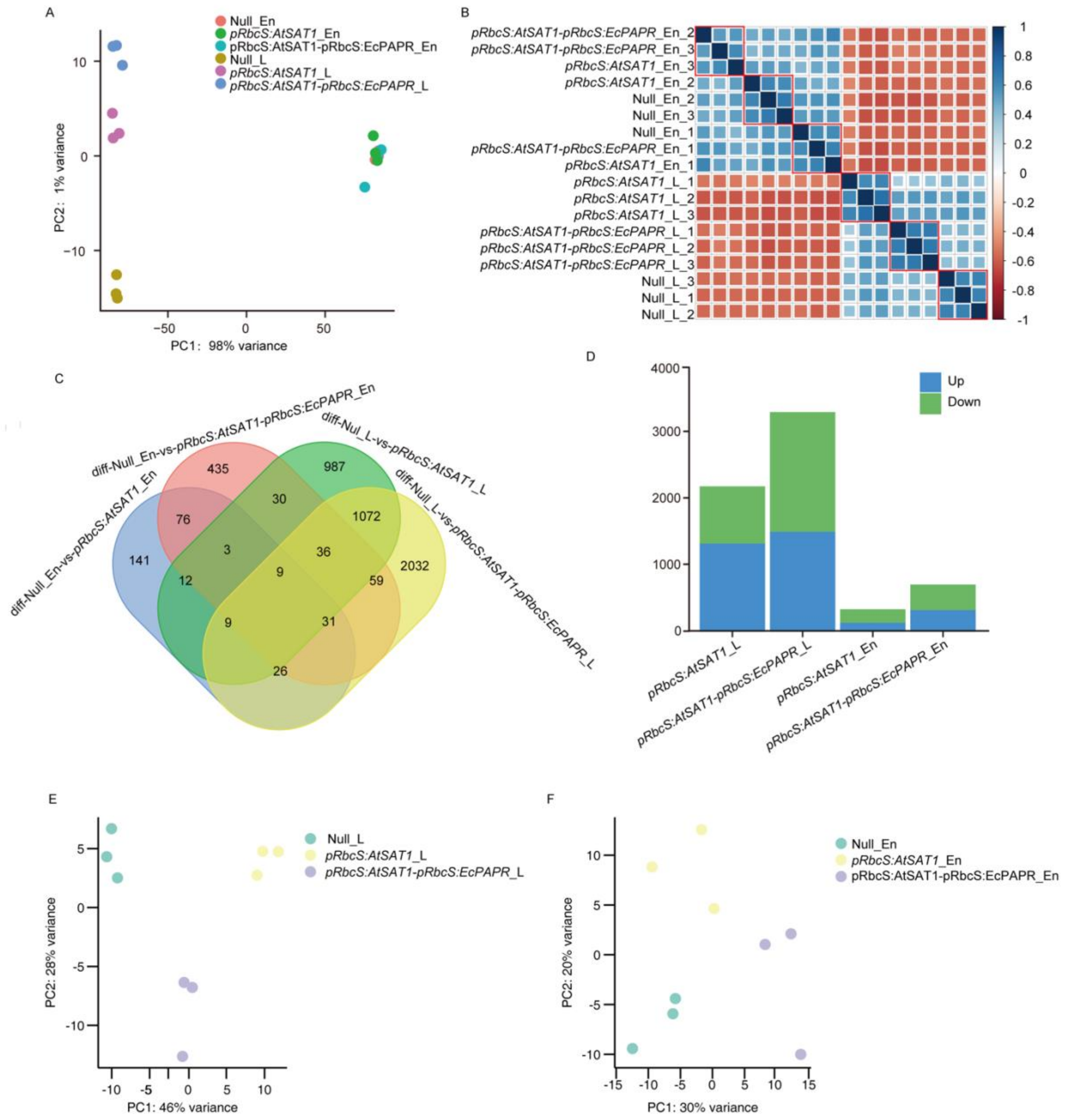

Figure S2 Global transcriptome analysis based on RNA-Seq data of sixth leaves of V9 stage maize plants and 18-DAP endosperms of Null, *pRbcS:AtSAT1* and *pRbcS:AtSAT1-pRbcS:EcPAPR*

(A) Principal component analysis of leaves and endosperms. (B) Hierarchical cluster analysis. The color scale represents the Pearson correlation. (C) Venn diagram exhibiting the number of expressed genes in the four genotypes. (D) The number of genes differentially expressed in the single and double mutants compared with Null. (E) Principal component analysis of leaves. (F) Principal component analysis of endosperms.

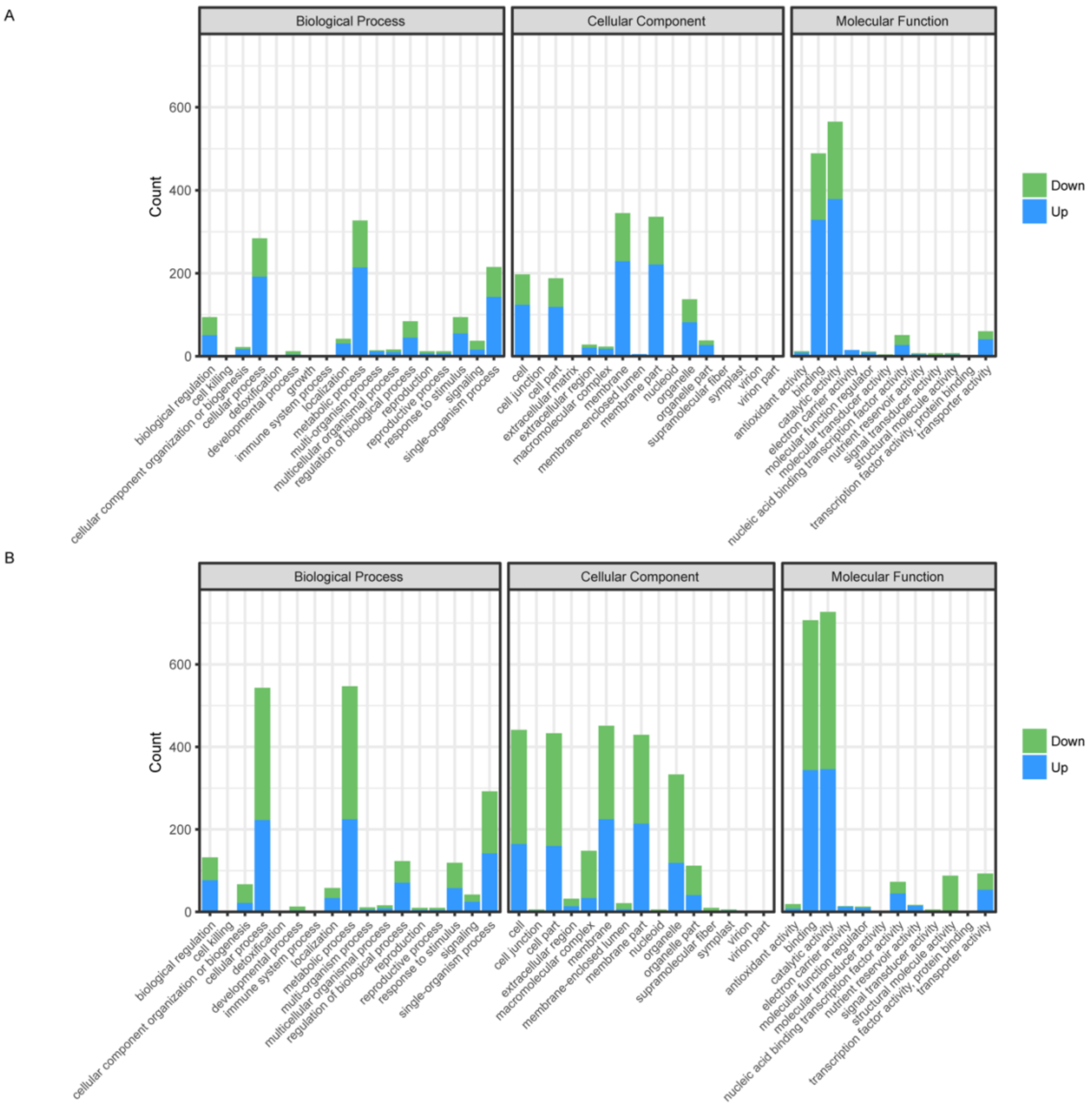

Figure S3 GO classification of DEGs in leaves

A, GO classification (corrected p-value < 0.05) of DEGs in *pRbcS:AtSAT1*_L vs Null_L. B, GO classification (corrected p-value < 0.05) of DEGs in *pRbcS:AtSAT1-pRbcS:EcPAPR*_L vs Null_L.

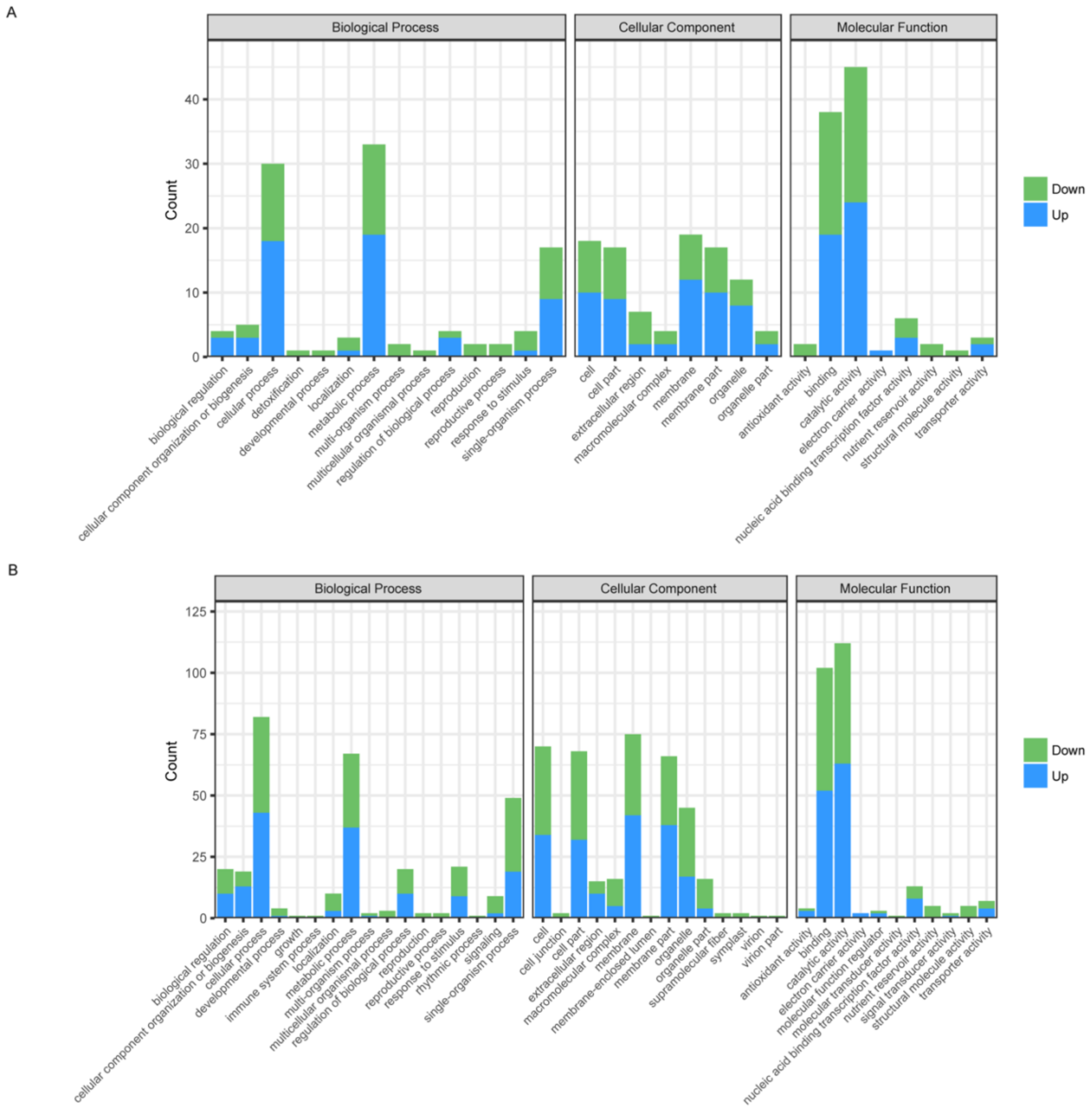

Figure S4 GO classification of DEGs in endosperm

A, GO classification (corrected p-value < 0.05) of DEGs in SAT1-OE_En vs Null_En. B, GO classification (corrected p-value < 0.05) of DEGs in *pRbcS:AtSAT1-pRbcS:EcPAPR*_ En vs Null_ En.

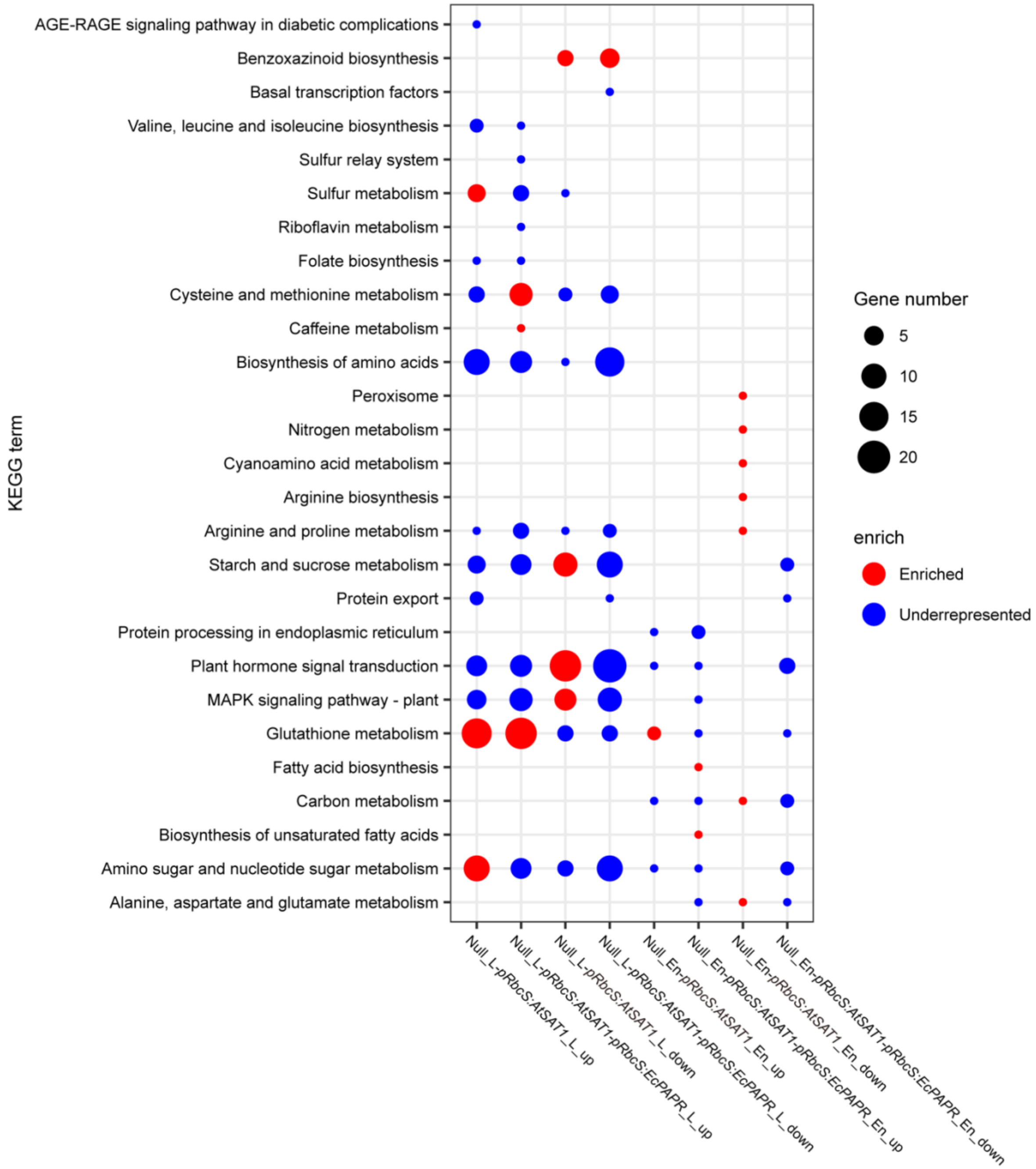

Figure S5 KEGG pathway classification of DEGs

The *y*-axis shows the KEGG pathway (corrected *p*-value < 0.05). The *x*-axis indicates the enriched factor. The point sizes describe the enriched numbers in each term. The red factors are enriched. The blue factors are underrepresented. (The figure was created by gg plot of the R language.)
